# MicroRNAs in Sepsis

**DOI:** 10.3390/biomedicines12092049

**Published:** 2024-09-09

**Authors:** Asimina Valsamaki, Vasileios Vazgiourakis, Konstantinos Mantzarlis, Rodopi Stamatiou, Demosthenes Makris

**Affiliations:** 1 Intensive Care Unit, Faculty of Medicine, University of Thessaly, 41500 Larissa, Greece; semi_val@hotmail.com (A.V.); vasvazg@yahoo.com (V.V.); mantzk@outlook.com (K.M.); dimomakris@uth.gr (D.M.); 2 School of Biology, Aristotle University of Thessaloniki, 54124 Thessaloniki, Greece

**Keywords:** sepsis, non-coding RNAs, microRNAs (miRs), inflammation, immune response, biomarkers, diagnosis

## Abstract

Sepsis is an insidious and frequent condition of severe inflammation due to infections. Several biomarkers have been established for initial screening, but the non-specific nature of the existing biomarkers has led to the investigation of more sensitive and specific tools, such as microRNAs (miRs). These non-coding RNAs are involved in several diseases, including sepsis, due to their roles in cellular homeostasis. Herein, a literature overview was attempted to distinguish the most prominent miRs identified in septic conditions and their usefulness in diagnosis, prognosis and even classification of sepsis. miRs implicated in the regulation of pro and anti-inflammatory mechanisms, such as MIR-146a, MIR-155, MIR-181b, MIR-223-5p, MIR-494-3p, MIR-2055b, MIR-150 and MIR-143 have been pinpointed as acceptable testing tools. Furthermore, the use of miRs as screening panels, specific for septic parameters, such as type of causal infection, inflammation immune pathways affected (NF-kB, STAT/JACK), organs inflicted, as well as parallel screening of certain miRs alongside other long non-coding RNAs (LNCs), as co-regulators of sepsis progression. Overall, miRs exhibit benefits in terms of specificity and sensitivity, as well as practical ease of use and test stability. Furthermore, miRs could offer valuable insights into the molecular basis of disease causality and provide valuable therapeutic information.

## 1. Introduction

According to the 3rd International Consensus [1], sepsis is redefined as a life-threatening condition due to dysregulated host responses to infection and the body’s own injury of tissues, which may lead to a septic shock produced by profound circulatory and cellular abnormalities. Therefore, sepsis is differentiated causally from systemic inflammatory response syndrome (SIRS) [2], which can present with the same clinical manifestations but without infection from microorganisms. In the same conference, the SIRS criteria of diagnosis were replaced with the SOFA score (sequential organ failure assessment), which is based on three necessary conditions to be present, that is, constant decreased blood pressure, increased breath rate, and change in consciousness—coma, delirium, lethargy, following infection [3]. Sepsis has been distinguished and differentiated from SIRS; therefore, it addresses only microbial infections. Yet most biomarkers are influenced by pre-existing conditions of the patient as well as possible comorbid effects. An ideal situation would be the coupling of the specific miR septic panel with other miRs that have been correlated to other disorders that might affect the patient’s endothelial and immune system response to the condition. Specific miR probes for many disorders are available and increase daily. Yet, it is important that further research should be directed into MIR-based comorbidity situations so that clinical application can become more efficient.

Epidemiologically, sepsis represents a major mortality cause, with an estimated annual death toll of over eleven million and a total of fifty million affected [4,5]. The mortality rate depends on the severity of sepsis upon detection, with severe conditions reaching 50%, and during septic shock, mortality could be as high as 80% [5]. Clinically, sepsis is characterized by multiple organ failure (MOF), including liver, kidney, CNS, respiratory, and cardiovascular systems and organs [6], and the amount of damage depends on the delicate equilibrium of pro vs. anti-inflammatory mechanisms [7]. The interval between onset and progression of sepsis is short; a complete diagnosis of the cause of infection, affected organs, and immune imbalance must be made promptly. The proposed strategy is to couple miR panels designed for specific conditions of sepsis alongside the testing of conventional biochemical markers. The major benefits of miR panels are their specificity, as explained, as well as the rapid results produced that can guide and limit further biochemical markers required. Several sets of such miR panels have been proposed herein with the potential to narrow down the type and cause of sepsis. Further, probes for several of the proposed miRs already exist, and biotech industries should be eager to easily design specific panels. These miR panels, once designed into functional kits, can produce results within an hour of testing blood samples, thus providing insights for sepsis sooner from the explosive progression of the disorder (6–12 h), as compared to testing for conventional biomarkers that require several hours [8]. 

Non-coding RNAs such as tRNAs and rRNAs have been identified from the early years of molecular biology, yet through time, different types of non-coding RNAs have been identified, with different nucleotide lengths, structures (linear or circular) and strandedness (single or double), puzzling researchers as to their functions, if any. Aided by the advances of next-generation sequencing techniques, genomics and transcriptomics, plenty of evidence indicates the epigenetic roles of these RNAs in cell function and tissue differentiation, as well as in pathological conditions, including cancer, immune system regulation and even neuronal mechanisms of thought processing. The basic classification of functional non-coding RNAs depends on nucleotide length, the long non-coding RNAs (LNC-RNAs) with lengths over 200 bases, and the micro RNAs with shorter lengths. Their complex biogenesis and mechanisms of maturation are indicative of assigned functions. LNC-RNAs have been shown to exhibit a competitive function with miRs acting as sponges de-activating miRs [9,10].

miRs, when processed to functional maturity via complicated intra-compartmental mechanisms, seem to exhibit specific functions assigned to them, usually by irreversible cleavage or decay of corresponding, complementary mRNA leading to translation inhibition [11]. Their role in the regulation of specific gene expression and vital cellular signaling pathways has been well established. Their biosynthesis varies largely, further indicating control mechanisms of their expression; for example, in *Drosophila melanogaster*, it has been estimated that miRs vary in rate of production from 17 to 200 molecules/min/cell [12]. The complicated pathways of functional maturation of miRs also imply checkpoint and control mechanisms since the initial premature hairpin loop molecule is processed into a single-stranded linear or circular functional molecule via the nuclear microprocessor system of DROSHA RNAse III coupled with cofactor DGCR8, transported via Exporter-5 to the cytosol for initial further processing from DICER protein, loading to the AGO Argonaut protein complex where double strands are separated, incorporated into the RISC (RNA-induced silencing complex) from where it is selectively released to act on its target mRNA [9,10]. Evidence of a highly controlled and compartmentalized mechanism, in essence, more complicated than the mRNA root to maturation and translation into polypeptide product, is being introduced. 

In this study, the role of miRs in sepsis, the underlying mechanisms of action, the targets regulated by their activity, and their potential prognostic and diagnostic value are examined.

## 2. Materials and Methods

For this review, the flexible guidelines of PRISMA (the PRISMA checklist can be found in the Supplementary data file) were followedThe method was based on a critical evaluation approach. All major databases were investigated in three consecutive phases of research, as follows:

PHASE I. Utilization of the keywords sepsis, non-coding RNAs, microRNAs, inflammation, immune response, biomarkers, and diagnosis using various combinations.
PHASE II. Further investigation is based on specific miRs of interest by their coded term, in combination with sepsis effects.
PHASE III. Evaluation of information obtained from thorough and recent review articles and cross-referencing of their sources.

**Figure 1:**
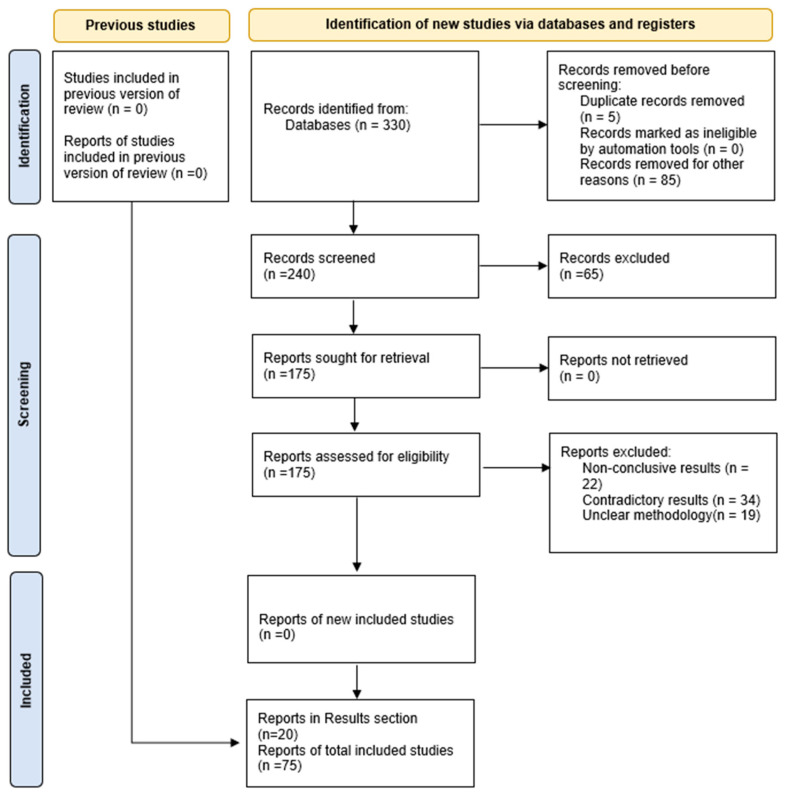
PRISMA 2020 flow diagram for updated systematic reviews, including searches of databases and registers only.

Given the vast amount of information retrieved (over 650 publications), further selection was performed using the standard inclusion and exclusion criteria suggested by PRISMA guidelines. The selected information was then further analyzed, encompassing works related to miRs with assigned functional roles in sepsis in correlation to specific tissue and organ origin, as well as to the corresponding effects on inflammatory pathways influenced by septic conditions. The correlation of miR function with other non-coding RNAs involved in a regulatory manner, as well as messenger RNA-induced inhibition of expression, resulted in the final outcome of the literature presented herein (Figure 1).

## 3. Results

It is apparent that valuable biomarkers are required for the efficient detection of sepsis due to its heterogeneity of the disease manifestation; thus, several significant molecular factors were used for reliable and early detection, as outlined in Table 1.

Biomarkers, like those in Table 1, have been utilized in diagnosing sepsis and evaluating the stage of the disease, while when combined, they are used to exclude or preclude causative pathogens, such as bacteria, viruses and fungi. Furthermore, they have been used as monitoring factors indicative of disease progress or inhibition. However, most of the used biomarkers are of a protein nature, and many of them are non-specific for sepsis but rather are indicative of the type of infection and the stage of the disease. There are also genomic and transcriptomic studies that can provide great insight into the whole genome and transcriptome [24]. Yet these are more quantitative rather than qualitative studies, and therefore, the need for more specific biomarkers directly related to the septic event that could provide useful insights with regards to the loss of balance of anti-inflammatory mechanisms that also lead to sepsis; researchers have been oriented in the investigation of the role of non-coding RNAs and especially micro-RNAs. 

The strong evidence supporting the ability of miRs to regulate protein expression at the post-transcriptional level led to extended research accompanied by clinical data in order to establish whether miRs could (a) be utilized as reliable prognostic and diagnostic biomarkers for sepsis and (b) whether the study of miR molecular functions could lead to the evaluation of direct relationships with the level of expression of inflammatory factors, thus procure a reliable treatment method for sepsis. 

### 3.1. Correlation of miR with Specific Sepsis-Related Cellular Functions

A thorough literature investigation revealed a vast amount of information pertaining to miR involvement in the septic process. Herein, recent research is examined based on disease state, specific tissue and miR-dependent cellular mechanisms, as outlined in Table 2. It has become evident that the function of miRs depends on several aspects, including tissue acting. In Table 2, cases of MIR-155 and MIR-181b refer to two studies performed on septic rat brains, with the one showing MIR-181b interference with NF-kB inflammatory pathway and detected down-regulation of the miR expression in hippocampus of septic rats [25], while the other study showed the same miR interfering with the JAK/STAT pathway and detected up-regulation of the miR in the cerebral cortex as well as in serum of septic rats [26]. Such differences were highlighted in a recent review [27]. Another miR, the MIR-494-3p, has been shown to interact with TLR-6 (toll-like receptor 6), disrupting the inflammatory pathway, and the same miR was found to be down-regulated in sepsis while TLR-6 was up-regulated [28]. The toll-like receptors are pathogen-recognition membrane molecules that recognize the so-called pathogen-associated molecular patterns (MAMPs) and activate pro-inflammatory responses via the NF-kB pathway and TNF-a and IL-6 secretion [29].

The MIR-574-5p has been shown to act on STAT1, decreasing its activity, and it is observed to be elevated in the serum of septic patients presenting decreased sepsis-induced AKI (acute kidney injury) [35]. Two other miRs, the 181-5p and the 2055b, seem to exert their positive effect in increasing anti-inflammatory response by down-regulating the expression of HMGB1 [36,38]. HMBG1 (high mobility group protein 1 or amphoterin) is an important nuclear protein that regulates the transcription of several vital inflammation-linked genes [46,47]. 

Additionally, it is interesting that certain miR down-regulation has been correlated with bacterial infections, such as MIR-96 for Gram-positive bacterial infections and MIR-101 for Gram-negative bacterial infections. Furthermore, MIR-150 has been shown to differ from controls in DNA virus infections causing sepsis [48]. Early identification of whether the causal bacteria is Gram-positive or Gram-negative via the proposed panel has an immense effect on selecting the correct antibiotic or other therapy much faster, even before microbial culture results are available. Discriminating the type of pathogen early on via the use of a MIR panel is essential for faster and more specific treatment of the septic patient. The identification of viral causes can redirect treatment into other therapeutic modes rather than the administration of antibiotics.

### 3.2. miR Diagnostic Value Evaluation Based on MIR Function

A recent well-structured and lengthy meta-analysis study [49] that employed 50 previous studies related to the investigation of miRs utilization in sepsis diagnosis, with totals of 5225 septic patients and 4008 controls, found that MIR-155-5p indicates reasonable diagnostic accuracy and the serum specimen to be reliable for clinical purposes, as shown by high sensitivity and specificity pooled values, while taking into account threshold effects and optimized cut-off standards in their data analysis. This conclusion partially contrasts a previous study where 22 studies with a total of 2210 septic patients and 1502 controls had pinpointed MIR-223-3p as the best candidate for reliable diagnosis of sepsis [50]. However, Zheng et al. assigned high values to the latter miR, even though it granted less reliability than MIR-155-5p. In essence, the two studies suggest at least two possible miRs in diagnosing sepsis condition.

Previously, it had been found that MIR-155-5p promotes malignant properties of proliferation, invasion and metastasis in colorectal carcinoma [51]. Chen et al. (2021) provided laboratory evidence that MIR-155 is strongly implicated in sepsis progression by enhancing inflammatory pathways [51]. It has long been suggested that MIR-155 targets mediators of inflammation such as TNF-a and interferons, compromising anti-inflammatory responses [52,53], while anti-inflammatory cytokine IL-10 seems to down-regulate MIR-155 [48,53]. Recently, Zeng Z et al. (2023) showed that the knock-down of MIR 155-5p expression rescues the suppressive effects on inflammatory responses of GAS5 [48]. GAS5 is an LNC that apparently sponges out the MIR-155-5p and reverses its proliferative pathogenic effects. Therefore, the theoretical background supports the notion that increased levels of MIR-155 are related to enhanced sepsis progression, and serum-soluble samples could easily be used for diagnostic purposes. Further, the GAS5 LNC could be attributed a therapeutic value by inhibiting MIR-155 [54].

In contrast to the negative role of MIR-155-5p, MIR-223-3p seems to function synergistically with anti-inflammatory defense mechanisms since it has been shown that in *Streptococcus*-based sepsis in mice, MIR-223 targets NLRP3 (NLR family pyrin domain containing 3) mRNAs and suppresses the formation of NLRP-3 inflammasome. Inhibition of MIR-223-3p causes the activation of NLRP3-based inflammatory pathways [34]. Further support for the notion of the protective role of MIR-223-p comes from a study analyzing blood samples and white blood cells of septic patients vs. controls, showing that expression of miR was inversely proportional to the rate of cellular apoptosis, an effect that was reversed with miR inhibitors [33]. The study provided experimental evidence that MIR-223 transfected Jurkat T lymphocytes exhibited enhanced proliferation and reduced apoptosis via augmentation of G1/S cell cycle transition by interacting and regulating FOXO1 activity. Considering the protective functions of MIR-223 in maintaining inflammatory response homeostasis during sepsis, one would expect the levels of MIR to be lower and not higher, as observed in the two meta-analysis studies above. However, controversy exists as to levels of MIR-223 detected, depending on the source (serum or plasma), organ affected and stage of disease [50].

MIR-150 is a good candidate for being utilized as a reliable sepsis biomarker, according to Formosa et al. (2022), since in eight out of nine studies, MIR-150 exhibited significantly lower levels of sepsis compared to controls. Ma et al.’s (2018) research [38] shows that miR protects cells from apoptosis by negatively regulating NF-kB1 inflammatory response. Yet another study confirms the notion of MIR-150 (and MIR-143) to be a reliable biomarker, as it was found to be down-regulated in sepsis T-cell immunoparalysis, correlating well with SOFA scores [39]. Formosa et al. also suggest that MIR-146a is a reliable biomarker, based on clinical and basic research data. More specifically, MIR-146a is down-regulated in septic children, even more during septic shock, and its levels correlate well with survival [55]. In a prior study, it was shown that MIR-146a seemed to specify sepsis compared to SIRS [56]. MIR-146a is expressed in almost all immune cell types and seems to control the NF-kB inflammatory pathway by direct regulation of TLR4 receptors [57].

Whole-genome analysis of critically ill septic patients revealed massive, up to 80% level, genomic alterations (genomic storm) in several cell types involved in sepsis [58]. Endothelial damage and, specifically, microvascular endothelial-cell dysfunction are considered hallmarks of sepsis and have been recognized as the principal causes of multi-organ failure [59,60]. Notably, these manifestations of sepsis have no known pharmacological or interventional treatment until now, thus imposing research on novel therapeutic targets. Sepsis-induced endothelial dysfunction was thoroughly reviewed by Ho et al. (2016), including studies in vitro, in vivo as well as clinical, either with experimental conditions resembling sepsis (e.g., LPS stimulation) or actual sepsis conditions at various stages of disease progression [60]. Several miRs were pointed out to exhibit a regulatory role associated with specific sepsis-related molecular mechanisms in endothelial tissue [61]. The most prominent observations involving miR down-regulation of endothelial activation were as follows:

- Inhibition of the slit2Robo4 pathway that regulates sepsis-induced endothelial activation is correlated with MIR-218 down-regulation. MIR-218 has been postulated to modulate endothelial inflammation [62];
- ADAMS15 disintegrin–metalloproteinase modulates endothelial permeability, and its mRNA expression has been shown to be inhibited by MIR-147b [45];
- MIRs-146a/b block components of pro-inflammation pathways such as NF-kB, AP-1 and MAPK [63];
- Angiopoietin-1 (AP-1) seems to be blocked also by MIR-150 [64];
- Inhibition of MIR-146a aggravates endothelial VCAM-1 expression [46].

Another clinical study investigated the role of claudin-2 (CLDN2) in vascular epithelial injury in septic patients. CLDN2 was shown to promote epithelial injury, while MIR-331 down-regulates CLDN2 levels in peripheral blood and restores cellular function [44].

Furthermore, several miRs have been shown to be overexpressed in sepsis-like induced conditions, affecting mechanisms that promote pulmonary inflammation such as JNK pathway and cholinergic stimulation, including MIR-194-3p, MIR-344a-3p, MIR-465-3p, MIR-501-5p, MIR-3596c, MIR-185-3p and MIR-877 [61]. Notably, MIR-127 was found to exhibit decreased expression at early septic stages, thus minimizing pulmonary injury since MIR-127, in normal conditions, activates JNK components [61].

A recent study, utilizing both in vitro human endothelial cells and an in vivo mouse model, provided strong evidence that ruscogenin exhibits considerable efficacy in sepsis treatment and blocks specifically MIR-146a-5p and, in parallel, activates NPR2 and SSH1 [65]. The results were confirmed when the reinduction of MIR-146a-5p reduced ruscogenin efficacy.

Since their discovery in 1997 by Asahara [66], endothelial progenitor cells (EPCs) have gained much attention on their role in vascular homeostasis and repair [67]. Besides this direct role, important functions have been attributed to this cell population by acting as paracrine mediators, releasing exosomes, thus mediating intercellular communication [68]. Notably, exosomes from EPCs contain molecules such as miRNAs that are transferred and captured by nearby endothelial cells, modulating their function at the post-transcriptional level [69]. Several miRs have been recognized as mediators of endothelial cell modulation in sepsis, such as MIR-126 [70]. More importantly, endothelial exosome-delivered MIR-126 protects from sepsis-induced cardiac dysfunction, a severe clinical manifestation of sepsis-induced organ damage, via regulating HSPA12B function [71]. These data support the additive hypothesis that endothelial progenitor cells and endothelial miRs may serve either as possible therapeutic targets in sepsis-related endothelial dysfunction or modulators of endothelial-cell function in sepsis by paracrine ways. 

Such studies highlight that the value of Rs as diagnostic biomarkers depends on several sets of conditions that may be taken into account in order for the MIR count to be a reliable and effective biomarker, with major considerations outlined as follows:

1. The inflammation mechanism involved in sepsis. Whether anti-inflammatory pathways are suppressed (MIR-181b, MIR-150 and MIR-223-5p) or pro-inflammatory pathways are augmented (MIR-155).
2. Identifying which organs are affected by sepsis since different miRs may be activated or silenced in different tissues and/or cell types, as discussed earlier with MIR-181b in different brain regions.
3. The age of the septic patient since, during differentiation and maturation, different miRs may be silenced or activated. For example, MIR-223 is shown to be up-regulated in pediatric sepsis, while MIR-146a is down-regulated.
4. The condition of the septic patient, e.g., MIR-141 is down-regulated in sepsis during pregnancy, and MIR-23b pinpoints disease severity [48].

Several other factors should also be considered since they modify expression levels of miRs regardless of disease state cellular response. miRs are also products of the genome, and their blueprints usually reside within the gene intronic regions or distant from the gene to be regulated (intra and intergenic biogenesis). In any case, gene products are susceptible to mutations that could alter their affinity for the target RNA, making it weaker or stronger. The miR genes could be silenced by mutations in regulatory regions or constantly turned on. Furthermore, miRs could be totally knocked down by excessive expression of corresponding sponging LNCs or by pitfalls of their export and maturation complicated mechanisms. Additionally, the causal factor for the miR expression in sepsis, as well as other diseases, needs to be determined. Whether the over- or under-regulation is a response to the pathologic cellular state or the result of pathogenesis also needs to be established. Given the firm experimental data that one of the basic functions of miRs is to regulate expression levels of corresponding complementary mRNAs, then in theory, there should be in a cell as many miRs as functional and structural proteins (or polypeptide subunits thereof) that is about 25,000 different miRs per differentiated cell of any tissue or organ. As a matter of fact, until 2017, over 2000 miRs have been identified, sequenced and classified [72]. Therefore, the abnormal levels of miRs observed in pathologic situations, such as sepsis, could only correspond to causative factors of the disease. Yet, this subject needs a lot more clarification, as well as cause and effect correlation.

### 3.3. Positive Aspects for the Utilization of miRs as Biomarkers for Sepsis

In the previous section, issues that may be limiting the usefulness of miRs as diagnostic biomarkers for sepsis, as well as other disorders (in the current state of knowledge as to their exact function), were discussed. Yet, even with current information available, miRs present several positive aspects of their utilization for screening, prognosis and diagnosis, as well as a direct or indirect guide to therapeutic venues. Some of the important factors that should be considered in using miRs to diagnose sepsis are as follows:

1. miRs have proven to be very stable and easily detectable in small samples of blood and blood fractions of serum, plasma, WBCs and any other cell component to be analyzed, as well as tissue and organ biopsies and aspirations.
2. Since each miR has been mapped to its specific mRNA target, the relevance with sepsis is associated with specific gene products known to regulate inflammation processes during the disease, thus validating the observed miR levels compared to healthy controls.
3. Since miR expression levels or silencing depend on cell and tissue differentiation, they can be used selectively as markers for age-dependent screening. For example, Fatmi et al. (2022) proposed a large set of 20 miRs as reliable biomarkers for neonatal sepsis based on increased or decreased levels of expression in sepsis compared to controls, all analyzed with the same RT-PCR technique [47].
4. Up or down-regulation of each suspect miR can easily correspond with inverse levels of target mRNAs and final protein products, confirming the validity of the observations correlating with specific cellular dysfunctions in the disease state.
5. miRs have been shown to be valuable tools in identifying or confirming the infection causative agent. For example, it has been shown that detectable consistent alterations of certain miRs are directly correlated with the source of infection, such as MIR-155 for DNA virus infections, MIR-96 for down-regulation in Gram (+) bacterial infections, and MIR-101 also down-regulated in Gram (−) bacterial infections [48].
6. miR expression levels can be used to monitor sepsis progression or attenuation; e.g., MIR-23b can be proposed as a biomarker for disease severity.

## 4. Discussion

Dysregulation of beneficial anti-inflammatory miRs indicates loss of homeostatic equilibrium and sepsis progression, whereas over-expression of the same miRs may be indicative of organisms’ response to restore homeostasis and disease recession. In contrast, pro-inflammatory miR over-expression could be indicative of disease progression, and their inhibition could be a cellular attempt toward homeostasis. If so, the rate of expression of targeted miRs should, in any case, be correlated with attenuation or progression of sepsis. 

Another aspect to be taken into account in order to maximize the usefulness of miRs in sepsis diagnosis and prediction, as well as exploring disease causality, is the association of miRs with the corresponding LNCs that competitively inhibit their activity. For example, a recent meticulous study demonstrated that MIR-27a-5p, which is involved in sepsis progression (more specifically, sepsis-induced lung injury), could act in inhibiting GSDMD mRNA, which promotes pro-inflammatory response by enhancing cytokine release and pyroptosis, and on the other hand, this miR is sponged out by LNC-ZNF33B-2.1, which regulates its activity [41]. Several other LNCs have been found to regulate corresponding miR targets, and therefore, miR activity could be compromised by LNCs, and the value of the miR could be lowered as detectable biomarkers. Therefore, the measurement of activity and expression of miRs alongside corresponding LNCs could be a valuable tool for a more reliable understanding of the epigenetic control mechanisms and produce more accurate prognostic and diagnostic results.

Given that a vast number of miRs have been identified to be correlated with sepsis onset, progression (or regression), organ inflicted, age of the patient and other parameters, as summarized in recent reviews [4,11,27,41,54], to mention just a few comprehensive and detailed studies, a depth of knowledge on suspect miRs exists as a reliable base of data for the utilization of miRs as diagnostic and screening tool for sepsis disorder. Even though there are studies published regarding the specific role of miR dysregulation in sepsis [4,54], the approach is either selective, namely only the most frequently identified miRs are discussed, or they are focused on some aspects of sepsis manifestation, for example, the immunological process. Herein, an attempt was made to distinguish from the vast amount of literature some of the most well-recognized miRs in sepsis, based on both preclinical and clinical studies. Furthermore, we aimed to associate these miRs with their specific target proteins and tissues affected, as well as the occurrence of miR dysregulation in disease states of progression or regression, in order to simplify the utility of miRs as diagnostic and screening tools. Endothelial tissue, a major target of septic damage, exemplifies the regulatory importance of miRs, as described earlier. 

In such an approach, it looks valuable to use miRs in sets in specifically oriented panels in order to enhance the magnitude and reliability of the results. The use of multiple miRs lowers the rate of interference from other factors that could dissolute the miR function, as discussed in detail earlier in the text. Additionally, the cumulative results could add extra reliability. Therefore, it is suggested that differentiated panels should be utilized, such as the following:

- The proposed panel for neonatal sepsis;
- Panel for confirming or even identifying sepsis causative infection;
- Panel for pro-inflammatory miRs and another for anti-inflammatory miRs;
- Panel for both miRs and corresponding competitive LNCs;
- Panel for detecting progression rate or disease attenuation;
- Panel for organ-specific miR functional dysregulation.

These more specific screening panels alongside conventional well-known but less specific protein biomarkers (as summarized in Table 1) could elicit much more valuable information regarding sepsis.

As shown in the results and Table 2, several miRs have been identified to be directly correlated with sepsis and the septic stage of the affected organism. Only MIR-2055b, which is correlated with late stages of sepsis, and MIR-23b, whose expression is directly correlated with disease severity, is mentioned. As explained throughout the text, the nature of sepsis depends on the type of infection, yet common regulatory endothelial and immune pathways are affected. 

Further, the coupling of miRs over-expressed (or down-regulated) in sepsis, alongside their corresponding regulatory (inhibitory) LNCs, could identify the exact pathway affected and even lead to therapeutic approaches. MIR-153 and GAS5 LNC, or MIR-27a-5p with LNC-ZNF33B-2.1, are already discussed. These miRs could be used to cancel corresponding MIR over-expression and sepsis progression if testing for these miRs indicates a significant change in expression. LNCs have already been tested as miR blockers in therapeutic applications, especially in lung cancers [73,74], as well as possible therapeutic tools in sepsis-induced organ dysfunction and reversal of inflammatory responses [72,75].

## 5. Conclusions

Through more than two decades of basic research and clinical evaluations, miRs have been shown to encompass a significant role in the molecular aspects of both pro and anti-inflammatory pathways in sepsis, thus acting as positive or negative monitors of disease progression/attenuation. This functional role justifies their utilization as sepsis biomarkers with high specificity. Furthermore, miRs seem to be valuable tools in identifying disease causality via their inhibitory interaction with specific complementary messenger RNA targets, thus acting as regulators of protein biosynthesis related to inflammatory cascade proteins.

Herein, routes to enhance the reliability and specificity of miR assays in septic conditions for diagnosis and even prognosis of the disorder are suggested. Furthermore, the functional roles of miRs can shed insights into the causality of sepsis and the organs and tissues inflicted. Lastly, it has been noticed that in most studies, miRs have been studied in isolation of mechanisms and molecular interactions that might alter their expression levels, thus rendering their biomarker value less reliable and consistent. A more holistic approach is suggested when studying miRs as biomarkers alongside corresponding LNCs and final messenger RNA protein products.

## Figures and Tables

**Table 1:** Well-known and utilized biomarkers for sepsis prognosis, diagnosis and identification of causative infection.

Biomarker	Role	Use	Reference
SuPAR—soluble urokinase-type plasminogen activator receptor	Non-specific inflammation marker, yet with prognostic value as high levels correlate with increased deaths	Prognosis	Backes 2012 [13]
PCT—procalcitonin	Distinguishes sepsis from SIRS	Diagnosis	Wacker 2013 [14]
sCD14—Presepsin	Macrophage/monocyte receptor recognizes LPS, useful for Gram(+) bacterial infections	Identification of cause	Kyriazopoulou 2023 [15]
Proenkephalin A	Reliable marker for progression of sepsis into acute kidney injury	Prognosis	Depret 2020 [16]
IP-10—interferon gamma-induced protein-10	Marker for progression into severe respiratory failure	Prognosis	Samaras 2023 [17]
CRP—C reactive protein	Frequently used marker for detection of bacterial infections, used in combination with IP-10, distinguishes between viral and bacterial infections	Identification of cause	Pool 2017 [18]
IL-6—Interleukin 6	Elevated levels correlate with severity of sepsis, especially in neonatal sepsis	Prognosis	Harbarth 2001 [19], Hou 2015 [20]
MCP-1—Monocyte Chemoattractant Protein-1	Cytokine with serum levels elevated in sepsis	Diagnosis	Holub 2018 [21]
sTREM-1—Soluble Triggering Receptor Expressed on Myeloid Cells-1	High sensitivity and specificity values in septic patients due to Shigella infection	Identification of cause	Huang 2019 [22]
CD64	Useful marker for early detection	Diagnosis	Wang 2012 [23]

**Table 2:** Specific miRs, most studied, selected to present the target and tissue specificity of the miRs and their either pro or anti-inflammatory properties. (Additional data for this table, such as population and age of people/animals studied, miRs source and the type of control groups used are presented as supplementary data).

MIR	Target	Tissue	Function	Reference
MIR-155		Myocardium and plasma	miR up-regulated in human sepsis	Vasques-Nóvoa F 2018 [30]
MIR-155	JACK/STAT pathway	Septic mice liver	Increased septic AHI	Lv-X 2015 [26]
MIR-181b	NF-kB pathway	Hippocampus	miR down-regulated in septic rats	Dong 2019 [25]
MIR-181b	sphingosine-1-phosphate receptor 1	Cerebral cortex and serum	Up-regulated miR in septic rats	Chen S-L 2020 [31]
MIR181-5p	HMGB1	Kidney of septic mice	Decreased infl response and decreased renal and hepatic dysfunction	Ma XF 2020 [32]
MIR-223-5p	FOXO1	White blood cells of septic patients	Inversely proportional expression with rate of apoptosis	Liu D 2020 [33]
MIR-223-5p	NLRP3	Septic mice	Suppresses formation of inflammasomes	Li 2022 [34]
MIR-494-3p	TLR-6		MIR lower levels, TOL6 increased levels	Wang H-F 2019 [28]
MIR-574-5p	STAT1	Increased in serum of septic patients	Decreased sepsis-induced AKI	Liu S 2021 [35]
MIR-2055b	HMGB1	Increased in serum and vital organs in septic mice	Increased a-infl activity in late sepsis	Zou 2016 [36]
MIR-23b	E-selectin, I-CAM1	Leukocytes	Expression correlated with disease severity	Zhou 2019 [37]
MIR-150	NF-kB1	HUVECs and sepsis mice	MIR over-expression protected from apoptosis	Ma Y 2018 [38]
MIR-150 and MIR 143	Nf-kB	Purified T cells from septic patients	Both miRs down-regulated in sepsis in correlation with SOFA scores	Mohnle 2018 [39]
MIR-146a	NF-kB		Down-regulated in sepsis and even more in septic shock	Karam 2019 [40]
MIR-27a-6p	GSDMD	Sepsis-induced lung injury	Sepsis progression rate of miR expression and miR regulation by LNC-ZNF33B-2.1	Lu Z 2022 [41]
MIR-96	NF-kB	Plasma fraction of septic neonatals	Down-regulated in sepsis produced by Gram (+) bacteria	Chen J 2014 [42]
MIR-101	PTGS-2	Plasma fraction of septic neonatals	Down-regulated in sepsis produced by Gram (−) bacteria	Chen J 2014 [42]
MIR-331	CLDN2	Peripheral blood of septic patients	Down-regulates CLDN2 activity and restores cellular function of endothelial cells	Kong 2020 [43]
MIR147b	ADAM15	Human vascular endothelial cells	Degrades ADAM15 mRNA as a protective mechanism	Chatterjee 2014 [44]
MIR146a	VCAM-1	mice	MIR-146a knock-down enhances inflammatory action of VCAM-1	Wu 2015 [45]

Abbreviations: AKI = acute kidney injury; FOXO1 = Forkhead box protein O1; GSDMD = Gasdemin D; HMGB1 = high mobility group protein 1; HUVECs = human umbilical vein endothelial cells; I-CAM = intercellular adhesion molecule 1; NF-kB = Nuclear Factor Kappa B; PTGS2 = prostaglandin-endoperoxide synthase 2; STAT1 = signal transducer activator of transcription 1; TOL6 = toll-like receptor 6.

## Data Availability

No new data were created.

## Supplementary Materials

The following supporting information can be downloaded at: https://www.mdpi.com/article/10.3390/biomedicines12092049/s1, Table S1. Specific miRs, most studied. Population and age of people/animals studied, miRs source and the type of control groups used are presented.
